# Improvement of participation rate in colorectal cancer (CRC) screening by training general practitioners in motivational interviewing (AmDepCCR)

**DOI:** 10.1186/s13063-022-06056-8

**Published:** 2022-02-14

**Authors:** Paul Aujoulat, Delphine Le Goff, Antoine Dany, Michel Robaskiewick, Jean Baptiste Nousbaum, Jeremy Derrienic, Mélanie Cariou, Morgane Guillou, Jean Yves Le Reste

**Affiliations:** Université de Bretagne Occidentale: Universite de Bretagne Occidentale, EA 7479 SPURBO, DUMG Brest, Brest, France

**Keywords:** CRC screening, Early detection, Motivational interviewing, Psychological approach, Professional-patient relations, Primary care, Study protocol

## Abstract

**Background:**

Colorectal cancer (CRC) is the second leading cause of cancer death in France (17,712 annual deaths). However, this cancer is preventable in the majority of cases by the early detection of adenomas. In France, the organized screening for CRC relies on general practitioners (GPs). The tests delivered by the GPs are carried out in 89% of cases. However, GPs do not systematically offer the test, because of time management and communication.

**Methods:**

AmDepCCR is a cluster randomized trial. Patients are prospectively included by their GPs. The study is designed in 2 phases for the GPs: first, GPs who have never participated in motivational interviewing (MI) training will be recruited then randomly split in 2 groups. Secondly, a 6-day motivational interviewing training will be carried out for the intervention group. Then, patients will be included in both groups during a period of 1 year. The primary outcome will be the number of CRC screenings achieved in each group and its difference. The secondary outcome will be the reluctance to screening and the patient’s self-estimated life expectancy at 0, 6, 12, and 24 months using the Health Belief Model (HBM).

**Discussion:**

This study will help to know if GPs motivational interviewing is useful to improve organized CRC screening. In addition, it may help to improve communication between patients and GPs. GPs will be able to improve their practice in other fields of application through motivational interviewing (other screenings, addictions…).

**Trial registration:**

2019-A01776-51 NCT04492215.

**Supplementary Information:**

The online version contains supplementary material available at 10.1186/s13063-022-06056-8.

## Administrative information

Note: the numbers in curly brackets in this protocol refer to SPIRIT checklist item numbers. The order of the items has been modified to group similar items (see http://www.equator-network.org/reporting-guidelines/spirit-2013-statement-defining-standard-protocol-items-for-clinical-trials/).

**Table:** 

Title {1}	Improvement of the participation rate in colo-rectal cancer (CRC) screening by training general practitioners in motivational interviewing (AmDepCCR)
Trial registration {2a and 2b}.	2019-A01776-51 https://www.clinicaltrials.gov/ct2/history/NCT04492215?A=1&B=1&C=merged#StudyPageTop
Protocol version {3}	V2.0 03/11/2020
Funding {4}	University of Western Brittany (Université de Bretagne occidentale – UBO)
Author details {5a}	Jean Yves Le Reste. MD, PhD, HDR, Professor of general practice. EA 7479 SPURBO. DUMG Brest. Paul AUJOULAT, MD. EA 7479 SPURBO. DUMG Brest. Michel Robaszkiewicz MD, PhD, Professor of Hepatogastroenterology. EA 7479 SPURBO, Director of the Finistere registry of digestive tumor. Jean-Baptiste Nousbaum MD, PhD, Professor, hepatogastroenterologist. EA 7479 SPURBO. Finistere registry of digestive tumor. Delphine Le Goff MD, MoS. EA 7479 SPURBO. DUMG Brest. Jeremy Derrienic. MD. MoS. EA 7479 SPURBO. DUMG Brest. Mélanie Cariou EA 7479 SPURBO. Coordinator-methodologist, Finistere registry of digestive tumor. Morgane Guillou MD, PhD. Psychatrist. EA 7479 SPURBO Antoine Dany, PhD. Public Heatlh Care, Epidemiologist. EA 7479 SPURBO Jerome Fonseca MD. EA 7479 SPURBO. DUMG Brest.
Name and contact information for the trial sponsor {5b}	Regional University Hospital Center of Brest Centre hospitalier regional et universitaire de Brest (CHRU) 2 Avenue Foch 29609 Brest Cedex
Role of sponsor {5c}	GPs randomization, data collection, data quality, data analysis.

## Introduction

### Background and rationale {6a}

Screening for colorectal cancer (CRC) is an important public health issue. Firstly, CRC is a common cancer worldwide: it is the third most common cancer in men and the second most common cancer in women. There are more than 1.2 million patients diagnosed with CRC and more than 600,000 deaths from the disease each year [1].

Secondly, the prognosis of CRC depends on its evolutionary stage at the time of discovery. The earlier the cancer is diagnosed, the better its prognosis will be. Because CRC often develops without symptoms or clinical signs at first, it is often diagnosed at later stages. Organized screening (OS) can detect CRC at an early stage of its development and detect lesions before cancer, allowing the disease to be treated. Thirdly, CRC most often develops from small, asymptomatic benign tumors called adenomas or adenomatous polyps. About 80% of CRC are estimated to occur from the transformation of adenomas. Some adenomas have a higher risk of malignant transformation (advanced adenomas) and it takes an average of 10 years for an adenoma to develop into a cancer [2].

There are different levels of risk for CRC among the population: 80% of CRCs are said to be sporadic because they occur without a particular context (average risk). Their frequency increases especially after 50 years, 15% of which are linked to a familial predisposition (personal or family history) or to pre-existing colon disease (high risk); 5% are linked to a genetic disease (very high risk). The prevention methods for CRC are adapted to this level of risk and the attending physician is the one who offers the prevention method that is adapted to each case. If someone belongs to the category of subjects at high or very high risk, they should be referred to a gastroenterologist offering to perform a colonoscopy as a first-line treatment depending on the level of risk and age. In case of very high risk, they will also be referred to a genetic consultation. The population at medium risk for CRC is the target of the French national organized screening program for CRC. This organized screening is intended for all people aged 50 to 74 years old who have no symptoms or particular risk factors. It consists of carrying out a test for blood in the stools every 2 years. This test can detect occult bleeding (not visible). Indeed, CRC or advanced adenomas can bleed intermittently and quietly and be responsible for invisible bleeding that can be detected by a stool test [2].

When CRC is detected at an early stage, the prognosis is good with more than 90% of survival rate at 5 years and the treatments used are less heavy, allowing a better quality of life. At the end of their first organized screening campaign for CRC, a study carried out by the Finistere digestive tumor register and the Association for cancer screening in Finistere (CRCDC) compared the characteristics of the lesions detected during 2060 colonoscopies performed after a positive test and 3794 colonoscopies performed as part of an individual screening [3]. A total of 827 adenocarcinomas of the colon and rectum were recorded. The diagnosis was made following a positive Hemoccult II® test for 212 cancers and following individual screenings for 54 cancers. The remaining 561 cancers are related to the non-responding population. The proportion of cancers with a good prognosis (UICC stage 0 to II) was higher in the context of organized screening (78,4%) and individual screening (80%), than in the non-responding population (57,2%) (*p*< 0.001). The yield of colonoscopy for advanced adenomas was higher in organized screening than in individual screening: 32 and 15 per 100 colonoscopies. This advance in diagnosis provided by screening resulted in a better prognosis of cancers which were diagnosed during screening (specific 5-year survival rate of 89%) than that of cancers diagnosed in the population that did not agree to invitations to the screening.

Until 2015 in France, organized screening for CRC was based on the Hemoccult test, the main criticism of which was its lack of sensitivity and the cumbersome nature of its implementation. The immunological test (OC Sensor) which replaced it, is a more reliable, easier-to-use test. It only requires a single stool sample (compared to 6 for the previous test). It can detect 2 to 2.5 times more cancers than the previous test and 3 to 4 times more polyps. Like any drug test, its reliability is not of 100%. An early cancer or a polyp may not bleed and may remain undetectable while the test is performed. These cancers that are diagnosed after a negative test are called “interval cancers.” They justify repeating the test every 2 years. These interval cancers have a better prognosis in people who get tested every 2 years than in those who do not screen regularly.

Organized screening takes place as follows: medium-risk subjects aged between 50 and 74 years are invited by mail, every 2 years, to consult their attending physician so that they can receive a screening test. The invitation letter is sent by the structure in charge of screening in each department (CRCDC for Finistere). The screening kit can also be given by the attending physician during a consultation. The GP then checks that their patient meets the eligibility criteria for organized screening (verification of the level of risk and the absence of symptoms or colonoscopy within 5 years) and directs them, if necessary, to the prevention modality which is adapted to their situation. This screening test is performed by the patient at home. It consists of taking a stool sample and sending it to a centralized medical biology laboratory in a prepaid envelope supplied with the kit containing the test. A detailed, illustrated user manual delivered with the test, facilitates its implementation. The laboratory transmits the result of the test within 15 days, with copies to the attending physician and to the management structure. If the result is negative (approximately 96% of cases), it means that no bleeding that could indicate the presence of cancer or precancerous lesions was detected at the time of the test. The test should be repeated every 2 years. If the result is positive (around 4% of cases), this does not necessarily mean that there is cancer, but merely that blood has been detected in the stool. To identify its origin, the GP refers their patients to a gastroenterologist who will perform a colonoscopy. As part of screening, colonoscopy detects a polyp in 30 to 40% of cases and cancer in 8% of cases. In more than half of the cases, it does not detect any anomalies.

The participation rate in organized screening for CRC (CRC OS) has been insufficient in France since its generalization in 2009 (32% on average) [4]. GPs play an important role in this screening because they are the main interlocutors to encourage target patients (50–74 years old) to participate. Thus, tests delivered by GPs are performed in 89% of cases by patients, unlike tests delivered by other means (pharmacists, relaunching of screening associations, etc.), which lead to the described average of 32%.

Therefore, the GP-patient therapeutic relationship is essential in the promotion of CRC OS and should be used to improve its coverage rate. A systematic review of the literature of recent studies conducted with GPs shows that training in communication (motivational interviewing) increases the number of tests performed from 11 to 12.2% [5–8]. There is also evidence of a stronger effect in women than in men for these interventions [9], which may lead to a gender analysis of the results. All of these data indicate that a GP-patient therapeutic relationship which is centered on the patient’s expectations makes it possible to cope with the resistance of an apparently healthy patient. It may help to perform a test that can lead to the diagnosis of a disease, with perhaps a gender difference.

GPs have already realized the value of motivational interviewing techniques. They understand its purpose, which is to enable behavioral changes in patients and to improve the process of shared medical decision-making [10]. Motivational interviewing is widely described and used as a technique that allows positive reinforcement of the patient and improves adherence and therapeutic alliance. Whether it is to stop drinking in social behavior disorders or addictions [11], smoking in the context of COPD, improving nutrition in the context of type 2 diabetes, or doing more physical exercise for cardiovascular diseases, these interventions are effective [12]. These change techniques are among their major daily concerns [13, 14] and meet their clinical needs. These are also at the heart of their core competencies, which promotes a holistic vision of the patient, and care centered on the patient’s needs. Membership of GPs is described and several continuing medical training organizations in France have developed training courses specifically dedicated to them.

This study will consider the effectiveness of motivational interviewing as a function of the level of resistance to screening, measured in patients at medium risk. It will be assessed by a patient self-questionnaire adapted from the Health Belief Model (HBM), which aims to predict healthy behaviors [15–17]. A repeated evaluation of the reluctance to screening and of the patient’s self-estimated life expectancy at 0, 6 months, 12 months, and 24 months will be carried out, allowing the calculation of a dynamic score to identify the evolution of perceptions of OS over 2 years.

The first CRC screening campaign in Finistere was followed at 48% by the target population. This rate fell during the second campaign in 2006 to 32% and subsequently remained at this threshold [18]. The CRC screening test is effective on patient mortality from a 45% participation, but a 65% participation is recommended to obtain an optimal decrease in mortality [19]. GPs play a central role in CRC screening by offering testing and repeating the individual and societal usefulness of screening for CRC. Thus, in 2015, a French study showed that when the tests were delivered by GPs, they were carried out in 89% of cases [20]. The main obstacle to the patient’s decision to perform the test was the absence of a proposal by the GP, and communication mainly focused on biomedical data [20]. Several studies have investigated the reasons why GPs do not offer the CRC screening test. It turns out that for GPs, it took too long to explain. Consequently, it was difficult to include CRC screening in a consultation.

Patients, for their part, expected an offer for the test from their GP and communication focused on their expectations [8]. Several national and international studies revealed that patients would like more communication centered around themselves for the presentation of the test [21]. Several studies focusing on communication and relationship training have shown that this allows an increase in the number of tests read. A study carried out in 2011 among 45 GPs in France showed a 12.2% increase in the number of tests performed. The participation rate of patients in the intervention group was 36.7% compared to 24.5% in the control group (*p* = 0.03), after the latter received a 4-h communication training in the part of CRC screening. This training consisted of 2 scenarios using a video on doctor-patient communication, one with a compliant patient and the other with a non-compliant patient, followed by a discussion and interactive methods including roleplay [8]. In 2013, a study that took place in the center of Southern France also showed an increase of 12% in performed tests. In this case, the GPs had attended 2 meetings. The first one approached screening tools and allowed for an exchange of practices between peers. The second one, 6 months later, allowed an analysis of changes in practices and introduced motivational interviewing techniques [5]. Other studies had shown more moderate increases in the screening rate, where interventions performed with GPs included doctor visits or educational workshops [6, 7, 22, 23].

Other researches have shown that prevention interventions focused only on education had no impact on opinion, motivation, or intention of target people and the effective screening rate [22, 24–27]. Consequently, these various studies seemed to indicate that interventions based on communication from the GPs, in particular while using motivational interviewing techniques, would be the most effective for increasing CRC screening rates. This is why this study offers to adapt GPs training in motivational interviewing to the promotion of colorectal screening.

### Objectives {7}

The main objective is to determine if training GPs in motivational interviewing is effective to increase the OS CRC participation rate of at least 10%.

The secondary outcome will be the reluctance to screening and the patient’s self-estimated life expectancy at 0, 6, 12, and 24 months using the Health Belief Model (HBM).

### Trial design {8}

This study is a cluster randomized control trial with GPs practice as a cluster unit. The allocation ratio is 1. It is a superiority trial. GPs agreeing to participate are randomized into control or intervention groups according to their CRC screening profile. GPs are divided in three screening profiles. Low screeners were defined as 0 to 19 screenings per year. Moderate screeners were defined as 20 to 100 screenings per year. High screeners were defined as more than 100 screenings per year.

The control group is composed of GPs who have never participated in motivational interviewing training. They include their patients for the study, without changing their practice with respect to CRC OS.

The intervention group also consists of GPs, similar to those in the control group, who never participated in motivational interviewing training.

In a health center with more than one GP, there could be GPs who benefited from motivational training and GPs who haven’t. This could result in cross-contamination. The authors think this will be limited because the GPs who have motivational training are asked not to talk about motivational interviewing to the other GPs during the study and because motivational interviewing requires some practice, which the GPs in the control group will not have at all. The intervention is not about learning what motivational interviewing is but about practicing and mastering it.

## Methods: Participants, interventions, and outcomes

### Study setting {9}

The study will take place in primary care in Brittany, France. The GPs will be contacted from the list of the County Council of the medical order of Finistère (890 registered in general medicine).

### Eligibility criteria {10}

Inclusion criteria for people who lend themselves to research:

- People at medium risk of CRC, residing in Finistère and aged 50 to 74 years old.
- The study applies to patients who have never participated in the organized screening of the organized screening management structure.

Criteria for not including people who are suitable for research:

- People who do not have an address allowing them to receive the HBM questionnaire are excluded from the study.
- People who do not meet the criteria for organized screening (high risk and very high risk of CRC).
- People who cannot give their written consent: patients under guardianship, curatorship, non-French speakers, illiterate people.

### Who will take informed consent? {26a}

Written consent will be obtained by GPs during the first consultation.

### Additional consent provisions for collection and use of participant data and biological specimens {26b}

On the consent form, participants will be asked if they agree to the use of their data should they choose to withdraw from the trial. Participants will also be asked for permission for the research team to share relevant data with people from the Universities taking part in the research or from regulatory authorities, when it is relevant. This trial does not involve collecting biological specimens for storage.

## Interventions

### Explanation for the choice of comparators {6b}

The comparators were chosen among GPs who had no motivational training since they fit in with the basic general practitioner population.

The data will be collected in both groups. The expected difference between the 2 groups is at least 10%. Indeed, the current screening rate is 35% in Finistère and its increase of 10% would make it possible to reach the minimum recommendations to influence mortality.

### Intervention description {11a}

The management of patients does not differ from their usual care. The offer to participate in this study will take place during one of their consultations at the GP’s office, not involving any additional time or travel constraints for the patient. Screening is offered to the entire target population by CRCDC.

GPs of the control group do not change their consultation method or their proposals around CRC screening.

During the study, the intervention group will receive a 6-day motivation interviewing training associated with video-call reminder sessions of 30 min. After the training, they will be allowed to include their patients.

Motivational interviewing training is provided by motivational interviewing experts and is part of the national university training organization in general medicine. The training takes place in person with all the investigators of the intervention group. The experts provide theoretical knowledge on motivational interviewing, but above all, practical training through the use of roleplays commonly used in motivational interviewing. This practical training is preferably focused on colorectal cancer screening in a person who has never participated in screening. There are 3 face-to-face 2-day sessions, spaced 3 months apart, in which all the intervention group participates. One month after the end of these 6 days of training, the participants of the intervention group meet in a group of 5 participants by videoconference with experts other than those of the initial training, belonging to the French-speaking association for the dissemination of motivational interviewing. This videoconference consists of the supervision of several consultations recorded in the interval by general practitioners in their daily practice.

At the end of the training, the participants in the intervention group should, in order to promote screening:

- Stick to the motivational spirit (Collaboration between two caregiver-patient experts/Acceptance of the patient’s value, autonomy, manifestation of empathy and valuation of their approach/Evocation of desires, capacities, reasons, and needs of the patient to change a behavior prejudicial to their health/Compassion which makes it possible to prioritize the well-being and the needs of their patients, in a spirit of benevolence).
- Acknowledge the temptation of the corrective reflex, realizing that dissonance is the fruit of the relationship
- Know the relational dead ends and how to “roll” with dissonance
- Have knowledge of the following basic skills: open questions, reflections, curriculum vitae, valuation, ask-share-ask, motivational balance, importance and trust scales
- Know the four fundamental processes: creating an alliance, focusing on a change objective, evocation, planning
- Know how to spot the speech change, or need change if necessary

Qualitatives studies by semi-structured interview are conducted before the start of motivational training to find out the barriers and facilitators encountered by GPs during colorectal cancer screening. Further qualitative semi-structured interview studies will be performed at the end of the study with GPs trained in motivational interviewing to find out how they used motivational interviewing during the study.

### Criteria for discontinuing or modifying allocated interventions {11b}

Subjects will be able to withdraw their consent and request to withdraw from the study at any time and for any reason. In the event of premature discharge, the investigator should document the reasons as fully as possible so that they can be analyzed. The investigator may temporarily or permanently discontinue a subject’s participation in the study for any reason that would best serve the subject’s interests, particularly in the event of serious adverse events, which is highly unlikely given the model of the study. In the event of a subject lost to follow-up, the investigator will make every effort to reconnect with the person. GPs who have agreed to participate will be able to withdraw from the study. A questionnaire will then be sent to the GP to find out the reasons for their withdrawal.

### Strategies to improve adherence to interventions {11c}

After the inclusion visit, during which patients have given their consent to participate, self-administered questionnaires at 6, 12, and 24 months will be sent to their homes.

On the same dates, the number of immunological tests for CRC performed will be noted.

If, at 24 months, the patient has not performed his immunological test, a telephone interview will be carried out in order to understand the reason for his refusal to perform this test. The analysis technique will be qualitative, using a thematical analysis. A verification of the completion of the questionnaires and the progress of recruitment will be carried out throughout the study.

Recruitment will be 1 to 2 patients per GP per month, for 12 months. This recruitment will be monitored and any delay in inclusion will be re-launched in both groups.

### Relevant concomitant care permitted or prohibited during the trial {11d}

Implementing motivational interviewing will not require alteration to usual care pathways (including use of any medication) and these will continue for both trial arms.

### Provisions for post-trial care {30}

The sponsor will ensure full compensation for the harmful consequences of research for the person who lends himself or herself to the study and its beneficiaries, unless they can prove the damage is not attributable to their fault or that of any party involved, without that may be accepted the act of a third party or the voluntary withdrawal or the person who had been consented to participate in the research.

### Outcomes {12}

The primary outcome of this study is to assess the impact of training general practitioners in motivational interviewing on the organized screening rate for CRC (CRC OS) at 24 months. The 24-month period varies and starts when individual patients are recruited.

The secondary outcomes are:

- Assessing the impact of training general practitioners in motivational interviewing, on the organized screening rate for CRC (CRC OS) at 12 months
- Exploring the ambivalence of patients with respect to the CRC OS by means of a self-questionnaire measuring the variables influencing health behaviors (health belief model) [28, 29] and exploring the opinion of patients on their life expectancy [30]. This assessment will take place at the start of the study, at 6 months, 12 months, and 24 months. It will be performed by all patients who have agreed to participate in the study.
- Exploring the reasons for patients who refused to take the test after 2 years.
- Identifying any difference in screening that could be linked to the patient’s gender, and quantifying this difference.

### Participant timeline {13}

**Fig. 1:**
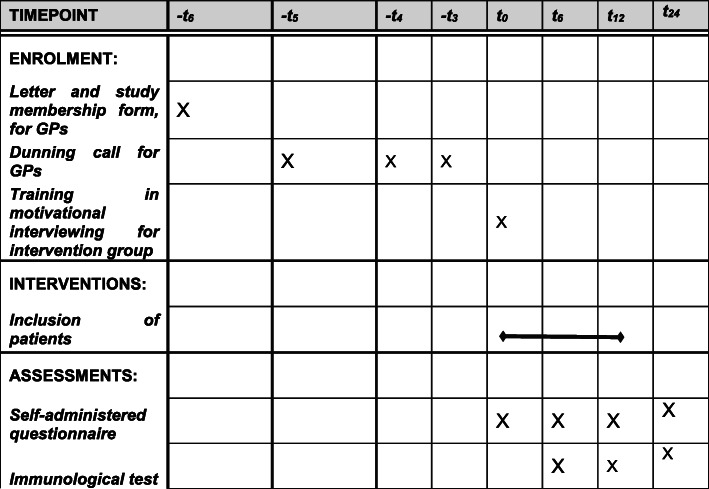
Enrolment, interventions, and assessments

The participant timeline is shown in Fig. 1.

### Sample size {14}

A classic randomized trial would require 752 subjects in total, for a power of 80%; an increase in the participation rate of 35 to 45%, and an alpha risk = 5% (two-tailed test). However, this power is here reduced by the existence of an intra-class correlation, measuring the degree of resemblance of the responses of patients from the same physician, quantified by the intra-class correlation coefficient ρ. The inflation factor by which to multiply the number of subjects is then (1+(m1) ρ), where m is the size of a cluster. The intra-class correlation coefficient (ICC) is typically of the order of 0.001 to 0.05. In the case of this study (CRC OS), an ICC value of 0.01 seemed reasonable. The correlation between doctors and patients is less important for screening procedures and the monitoring of acute pathologies than it is for the monitoring of chronic pathologies. With 82 GPs including patients and an average cluster size of 10 assessed patients per GP, the chosen coefficient is 1.09. A total staff of 820 patients divided between 82 doctors, will guarantee a power of 80%. With 5% lost to follow-up, the number of patients needed is 864 patients, which will be rounded up to 902, or 11 patients per GP to compensate for loss.

The software used for the statistical analysis will be R and SAS software, depending on the analysis.

### Recruitment {15}

A total of 902 patients will have to be recruited, or 451 patients per arm. In each arm, 41 GPs will need to recruit an average of 11 patients over a period of 1 year. This represents 1 to 2 recruitments per month per GP.

In Finistere, the medical density of GPs is 113 doctors per 100,000 inhabitants, or 885 patients per GP (STATISS 2015, ARS Bretagne), which ensures the feasibility of the study.

General practitioners recruit during their usual consultations. When a patient meets the eligibility criteria to join the study, the GP presents the study to him and suggests that they enter the protocol.

Recruitment is facilitated by patients who receive the CRC OS screening introductory letter usually sent during the national CRC OS promotion campaign, and who may mention it to their GP.

## Assignment of interventions: Allocation

### Sequence generation {16a}

GPs in the control and intervention group will be randomized. GPs who have received training in motivational interviewing in the past will be excluded. The stratification will make it possible to obtain 2 homogeneous groups of physicians in terms of the participation rate of their patients in screening the year preceding the study. This randomization will be carried out using groups of screening test prescribers defined by the Association for cancer screening in Finistere (CRCDC). GPs are divided into three screening profiles. Low screeners were defined as 0 to 19 screenings per year. Moderate screeners were defined as 20 to 100 screenings per year. High screeners were defined as more than 100 screenings per year For each doctor successively included in the trial, the randomization list will give his or her status as participating or not participating in the MI training. The allocation ratio is 1.

### Concealment mechanism {16b}

The allocation sequence is performed by opaque sealed envelopes, randomized in 1:1, stratified on the screening profile.

### Implementation {16c}

The recruitment of GPs will be carried out by the authors. It will be done initially by mail, followed by 3 reminders every month by email or by phone. These GPs will be contacted from the list of the Finistère order of doctors. GPs who say they have already been trained in motivational interviewing are excluded. Randomization will be carried out by the data management unit of the CHRU of Brest.

## Assignment of interventions: Blinding

### Who will be blinded {17a}

As the study is based on the participation of GPs in training, the trial is necessarily open to physicians. However, patients will not know if their doctor has received training in motivational interviewing and will be assigned randomly in groups. The outcome assessment is objectively measured electronically.

### Procedure for unblinding if needed {17b}

Only outcome assessors and patients are blinded so unblinding will not occur.

## Data collection and management

### Plans to promote participant retention and complete follow-up {18b}

No compensation is provided for patients.

### Data management {19}

All the information required by the protocol will be recorded in an electronic case report form (CRF). The data will be disseminated as and when they are obtained, and explicitly recorded in these notebooks. Each missing item must be coded. The online filling of the observation notebook by the investigator allows the data to be viewed quickly and remotely. The investigator is responsible for the accuracy, quality, and relevance of all data entered. In addition, when entered, this data is immediately verified through consistency checks. As such, it will validate any change in value in the CRF. These modifications are subject to an audit trail. A justification can be included as a comment. A paper printout will be requested at the end of the study, authenticated (dated and signed) by the investigator. A copy of the authenticated document intended for the sponsor will be archived by the investigator.

Data entries will be carried out electronically via an internet browser. The setting of the CRF as well as the data management of the study data will be carried out by the data management unit (DMU) of the CHRU of Brest using the Ennov Clinical software®. The data analysis will be carried out by the statistical service of the DMU of the CHRU of Brest using SAS and R software. The CRCDC data extraction (i.e., has the screening been carried out or not) will be done on their business software, Lynx®, thanks to the IT services company in charge of the development of this tool. The data required for the study will be integrated into the clinical database using the CS Import module of the Ennov Clinical software.

### Confidentiality {27}

This study requires access to data from directly identifying medico-administrative databases (name, maiden name, first name, date of birth, welfare number, affiliation fund, attending physician, postal address, telephone, date of last update), the “reference methodology” (MR-OO1) in the application of the provisions of article 54 paragraph 5 of the law n°78-17 of January 6, 1978, as amended relating to data processing, files and to freedoms does not apply, and it is necessary to apply for authorization from the *Commission Nationale de l’Informatique et des libertés* (CNIL) in order to be able to access this identifying data. The data processing also complies with the General Data Protection Regulation of the European Union No. 2016/679.

In accordance with the provisions concerning the confidentiality of data to which the persons responsible for the quality control of research have access (Article L.1121-3 of the Public Health Code), in accordance with the provisions relating to the confidentiality of information, in particular related to the nature of the products, the tests, the people who are suitable for them and the results obtained (article R.5121-13 of the public health code), the people with direct access will take all the necessary precautions to ensure confidential information relating to the products, the test, the persons who are suitable for them and particularly regarding their identity and the obtained results. These people, as the investigators themselves, are subjected to professional secrecy (under the conditions defined by articles 226-13 and 226-14 of the penal code). During or at the end of the research, the data collected on suitable people and sent to the sponsor by the investigators (or any other specialized stakeholders) will be coded. They must not in any case show the names of the persons concerned or their addresses in clear. Only the first letter of the subject’s last name and first name will be recorded, along with a study-specific coded number indicating the order of inclusion of subjects. The sponsor will ensure that each person who is involved in the research has given their written consent for access to the individual data concerning them and is strictly necessary for the quality control of the research.

### Plans for collection, laboratory evaluation, and storage of biological specimens for genetic or molecular analysis in this trial/future use {33}

There will be no biological specimens collected.

## Statistical methods

### Statistical methods for primary and secondary outcomes {20a}

A descriptive analysis of GPs and recruited patients will be carried out using the usual statistical parameters: frequency and percentage for qualitative variables; number, mean, standard deviation, median, quartiles, minimum, and maximum for quantitative variables. The comparison of response rated between the two groups takes into account the within-class correlation indicated by cluster randomization. A GEE logistic model (Generalized Estimating Equations, Liang & Zeger, 1986) will be used, with the “sandwich” estimator of the variance matrix. The GEE method requires retaining the structure of the correlation matrix describing the similarity of response within the same cluster. For cluster randomized trials, it is natural to assume that this matrix is “swappable” in shape. This modeling can obtain an odds ratio with a confidence interval not biased by the existence of an intra-class correlation. The analysis of the secondary criteria used, for the same reasons, a mixed linear model making it possible to take into account the intra-class correlation using an “exchangeable” structural correlation matrix. It is an intention-to-treat analysis.

For people who have not completed their 24-month test, open individual telephone interviews will be conducted to explore the reasons.

### Interim analyses {21b}

The interim analyses will follow the same rules as those described previously. The interim analyses will concern the analyses at 6 and 12 months. There are no anticipated problems that are detrimental to the participant.

### Methods for additional analyses (e.g., subgroup analyses) {20b}

There will be no additional analyses.

### Methods in analysis to handle protocol non-adherence and any statistical methods to handle missing data {20c}

Missing data will be identified by a clinical researcher from the Ea SPURBO of Brest. Investigators will be called back to complete the data. The remaining incomplete files will be declared as lost to follow-up.

### Plans to give access to the full protocol, participant level-data and statistical code {31c}

All plans and anonymized data will be accessible from an electronic drive. Access to the data can be obtained on request.

## Oversight and monitoring

### Composition of the coordinating center and trial steering committee {5d}

The coordinating center will be provided by the delegation of clinical research and innovation of the CHRU of Brest. It will ensure the proper conduct of the study, the collection of the data generated in writing, their documentation, recording, and report, in accordance with the Standard Operating Procedures implemented within the CHRU of Brest and in accordance with Good Clinical Practices as well as the legislative and regulatory provisions in force.

Information regarding participation in the CRC OS will be collected at the organizational center, CRCDC, which ensures that information is collected in the same way for all patients

### Composition of the data monitoring committee, its role and reporting structure {21a}

The investigator and the members of his team agree to make themselves available during Quality Control visits carried out at regular intervals by the Clinical Research Associate. During these visits, the following elements may be reviewed in accordance with the monitoring grade defined by the sponsor for the study: informed consent, compliance with the study protocol and the procedures defined therein, quality of the data collected in the observation notebook: accuracy, missing data, consistency of the data with the “source” documents (self-questionnaires)

### Adverse event reporting and harms {22}

As part of this study, the investigators will notify the adverse effects in relation to the research occurring during the participation of the patients via a declaration form, in order to allow the constitution of a register which will be used for the drafting of the final report of the study. The risks likely to expose the person who is lending themselves to the research are trivial, and the examinations present very few undesirable effects; they are neither painful nor physically restrictive. The psychological constraints incurred by the person are those relating to the CRC OS, that is to say an apprehension linked to an expectation of a result. Research does not increase this risk because screening is designed to be done by all people at average risk of developing CRC and the training offered to the GPs aimed at improving communication by answering patient questions, which will lead to a reduction in psychological constraints.

### Frequency and plans for auditing trial conduct {23}

The investigators undertake to accept the quality assurance audits carried out by the sponsor. All data, documents, and reports may be subjected to audits and regulatory inspections.

### Plans for communicating important protocol amendments to relevant parties (e.g. trial participants, ethical committees) {25}

In the event of a substantial modification made to the protocol by the investigator, it will be noticed and approved by the sponsor. The latter must obtain a favorable opinion from the institutional review board (IRB) prior to its implementation. A new consent from people participating in the research will be obtained if necessary. If necessary, the protocol will be updated in the clinical trial registry.

### Dissemination plans {31a}

The scientific communications and reports corresponding to this study will be produced under the responsibility of the principal investigator coordinating the study with the agreement of the responsible investigators. Co-authors of the report and publications will be the investigators and clinicians used, in proportion to their contribution to the study, as well as the biostatistician and associated investigators.

## Discussion

If the intervention increases the screening rate by 10%, this would allow the minimum recommendations to be reached in order to influence mortality. Since the procedures for screening and medical training are the same throughout France, this would involve training all future GPs in motivational interviewing techniques. GPs could also be trained through continuing education. As motivational interviewing techniques do not differ depending on the language, these results could be extended to countries where there is organized screening for colorectal cancer. Colorectal cancer screening is not the only one that is nationally organized in France. Motivational interviewing techniques could be extended to breast cancer screening and cervical cancer screening, two cancers for which there is already nationally organized screening in France. In addition, motivational interviewing techniques can be used in areas other than cancer screening. It has been shown to be effective in alcohol use disorders, smoking, or diabetes, which are part of GPs core competencies. This would therefore be one more argument for training GPs in motivational interviewing, and therefore have positive repercussions on various health problems found in primary care.

## Trial status

V2.0 03/11/2020. Recruitment began in June 2021. The end of recruitment is estimated at June 2022.

## Supplementary Information

**Additional file 1.**

## Abbreviations

AmDepCCR: *Amélioration du dépistage du Cancer Colorectal*
CHRU: *Centre Hospitalier Régional Universitaire*
CNIL: *Commission Nationale de l’Informatioque et des Libertés*
COPD: Chronic obstructive pulmonary disease
CRC: Colorectal cancer
CRCDC: *Centre Régional de Coordination des Dépistages des Cancers*
CRC OS: CRC organized screening
CRF: Case report form
DMU: Data management unit
EA SPURBO: *Equipe d’Accueil Soins Primaire, Santé Publique, registre des cancers de Bretagne Occidentale*
GEE: Generalized Estimating Equation
GP: General Practicioner
HBM: Health Belief Model
ICC: Intra-class correlation coefficient
IRB: Institutional review board
MI: Motivational Interviewing
OS: Organized screening
T0: Time 0
T6: Time 6 months
T12: Time 12 months
T24: Time 24 months
UICC: Union for International Cancer Control
