# Supplementary figures and images for: Improvement of participation rate in colorectal cancer (CRC) screening by training general practitioners in motivational interviewing (AmDepCCR)
Source: Trials. 2022 Feb 14;23:144. doi: 10.1186/s13063-022-06056-8 (PMC8842548; doi:10.1186/s13063-022-06056-8)

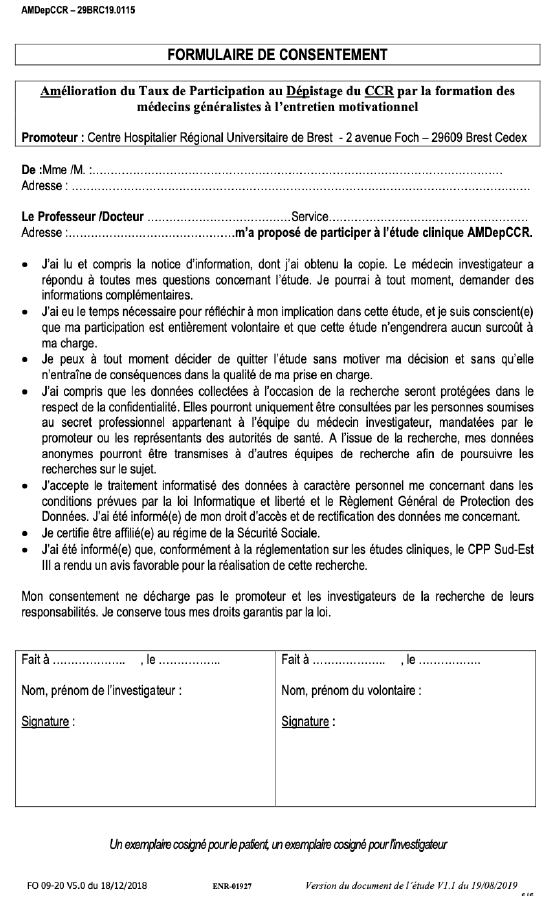

Supplement: Supplementary file 1 — Additional file 1. [file 13063_2022_6056_MOESM1_ESM.png]
